# Postsurgical perilesional functional connectivity predicts neurological outcome in glioma patients

**DOI:** 10.3389/fnins.2026.1751746

**Published:** 2026-02-05

**Authors:** Derek Madden, Alissa J. Schroeder, Mingwei Huang, Tressie M. Stephens, Lei Ding, Ian F. Dunn, Han Yuan

**Affiliations:** 1 Stephenson School of Biomedical Engineering, Gallogly College of Engineering, The University of Oklahoma, Norman, OK, United States; 2 Department of Neurosurgery, The University of Oklahoma Health Sciences Center, Oklahoma City, OK, United States; 3 Institute for Biomedical Engineering, Science, and Technology, University of Oklahoma, Norman, OK, United States

**Keywords:** glioma, functional magnetic resonance imaging, neurological outcome, overall survival, prediction, resting state functional connectivity

## Abstract

**Introduction:**

The study investigated glioma patients after surgical resection of tumor tissue using postoperative functional magnetic resonance imaging (fMRI) to assess cavity-adjacent (perilesional) functional connectivity as a predictor of overall survival and functional recovery.

**Methods:**

We developed an analytic method to quantify the postoperative whole-brain functional connectivity. Resting-state whole-brain fMRI scans acquired from 12 glioma patients following surgical resection were analyzed as part of a proof-of-concept study. In particular, connectivity of the resected perilesional area was compared to that of the corresponding contralateral homologue region, and the difference between perilesional and contralateral connectivity was calculated. To test whether the functional connectivity metric could predict recovery of neurological outcomes, we compared patients’ connectivity metrics from postoperative scans with changes in Karnofsky Performance Status (KPS) score between preoperative assessment and 6-month follow-up. Additionally, we examined whether the connectivity metric could predict overall survival by dividing the patients into subgroups based on their median survival time and comparing connectivity metrics.

**Results:**

Our analysis showed altered functional connectivity between perilesional and corresponding contralateral regions following surgical resection of glioma. The connectivity metric from postoperative scans was significantly correlated with recovery of neurological outcomes, as reflected by changes in KPS from preoperative to 6 months postoperative period (*ρ* = 0.97, *p* < 0.001). Moreover, individuals with survival times greater than 15 months showed significantly higher connectivity than those with shorter survival times (*p* = 0.0016 and Cohen’s *d* = 2.74 in all subjects, *p* = 0.02 and Cohen’s *d* = 1.90 in the subset of subjects with Grade IV gliomas). Furthermore, we developed machine learning models based on functional connectivity features, and they were able to predict the survival time with an accuracy of 92% and predict the KPS changes with an absolute error of 5.84 ± 6.08.

**Discussion:**

Overall, our study showed that resting-state fMRI from patients after glioma resection is relevant to their long-term neurological outcomes: decreased connectivity in the perilesional regions compared to the contralateral regions indicates less survival time and worsened functional outcomes. The reported analytics from postsurgical fMRI scans, combined with the machine learning model, could provide important prognostic information for postsurgical recovery management.

## 1 Introduction

Gliomas are the most common form of intrinsic primary tumor in the human brain (Sanai and Berger, 2018). Malignant brain tumors (i.e., brain cancer) are among the deadliest cancer types—estimated to be the 9th leading cause of cancer death in 2025 for both males and females in all age groups (Siegel et al., 2025). Surgical resection is the current mainstream treatment for gliomas, especially those of high grade (Delgado-López and Corrales-García, 2016; Sanai and Berger, 2018). Despite progress in diagnosis and treatment strategies, the overall survival rate is still poor: the 5-year relative survival rate in malignant brain tumors is only 35.6% (Siegel et al., 2025).

The surgical resection mostly impacts the oncologic prognosis of gliomas and is variable based on the clinical characterization (Buckner, 2003; Hervey-Jumper and Berger, 2014; Zigiotto et al., 2020). Within surgery, a greater extent of resection (EOR) is associated with enhanced survival (Chaichana et al., 2014; Sanai and Berger, 2018). However, greater resection can be both difficult and dangerous due to the neurological deficits that may arise from the removal of brain tissue, given the infiltrative nature of the tumors into the surrounding brain (Rahman et al., 2017). Consequently, there is an urgent need to formulate more accurate oncological prognoses, especially considering both survival and neurological recovery, to improve the balance between effective resections and functional preservation (Hervey-Jumper and Berger, 2016).

Moreover, it is increasingly evident and acknowledged that the functional anatomy of the human neocortex is plastic and capable of reorganization (Cargnelutti et al., 2020; Herbet et al., 2024). A large proportion of postoperative short-term deficits (60–80%) observed at 1 week after surgery recover at long-term follow-ups at 3 or 6 months after surgery (Tarapore et al., 2012). However, the ability to recover and the extent of recovery vary dramatically from patient to patient. Thus, predicting plasticity is crucial for building an oncological prognosis to support individualized treatment and management.

Our current study investigated the ability of functional magnetic resonance imaging (fMRI) to provide prognostic information following surgical resections of gliomas. We constructed our study by collecting the scans within 3 days after the surgical resection of gliomas and compared the images to the survival time of the patients, as well as the neurological examinations before the surgery and 6 months after surgery. In particular, whole-brain fMRI scans were acquired, thereby enabling mapping of brain-wide functional connectivity. Furthermore, patients were scanned in a resting state, which did not require them to perform tasks that could be difficult for those already with certain neurological deficits. Many prior studies have studied presurgical fMRI and suggested it as a promising tool to map eloquent brain regions before a surgical procedure (Fandino et al., 1999; Mueller et al., 1996). fMRI for presurgical planning is more oriented toward specific sensorimotor or language regions in the brain (Vlieger et al., 2004). However, this study examined postsurgical fMRI scans acquired within 3 days of surgery prior to the patients’ discharge, to support oncological prognosis. Thus, we hypothesize that the functional connectivity between regional brain tissues and at the level of whole-brain networks, as captured by resting-state fMRI, can predict survival time and recovery of neurological function. We developed an analytic method to quantify the postoperative whole-brain functional connectivity and tested our hypotheses.

## 2 Methods

### 2.1 Subjects

This study was performed in accordance with the Declaration of Helsinki. This study protocol was approved by the Institutional Review Boards at the University of Oklahoma Health Sciences Center (OUHSC). Subjects were recruited as patients who underwent surgical resection of gliomas of any World Health Organization (WHO) grade. Subject inclusion was entirely voluntary, and care continued as normal for subjects who elected to participate. All adult participants provided written informed consent to participate in this study.

### 2.2 MRI acquisition

Magnetic resonance imaging (MRI) data were acquired using a 1.5 Tesla (1.5 T) MR450w (GE Healthcare, Milwaukee, Wisconsin) MRI scanner at OUHSC. Resting-state fMRI data were acquired with an echo-planar imaging (EPI) sequence (repetition time (TR)/echo time (TE) = 2,400 ms, field of view (FOV)/slice thickness = 240/4 mm, acquisition matrix = 64 × 64, voxel size = 3.75 mm × 3.75 mm × 4 mm). All subjects were instructed to rest with eyes closed and to remain still. A total of 240 volumes were collected for the resting state, which lasted 9.6 min. T1 Magnetization-Prepared Rapid Gradient-Echo datasets were also obtained for anatomical analysis (TR/TE = 8.82/3.46 ms, acquisition matrix = 256 × 256, FOV/slice thickness = 240/0.8 mm, voxel size = 0.9375 mm × 0.9375 mm × 0.8 mm). All scans were acquired within 3 days following the surgical resection.

### 2.3 Clinical evaluation

Karnofsky Performance Status (KPS) score was evaluated for all subjects preoperatively and again at 6 months postoperatively to gather information on cognitive function before resection and throughout the process of recovery. The KPS is a standard tool for quantifying brain tumor patients’ general wellbeing and activities of daily life (Sacko et al., 2015). Performance status is an assessment of functional impairment, and KPS scores were obtained at the aforementioned points to evaluate long-term functional changes beyond surgical resection.

### 2.4 fMRI data preprocessing

fMRI Data preprocessing was conducted using customized Analysis of Functional Neuroimages (AFNI) scripts following our prior protocol (Huang et al., 2025; Madden et al., 2025; Yuan et al., 2014; Zhang et al., 2023). The first five volumes of each run were excluded to allow the signal to reach a steady state. Key processing steps were bandpass filtering (0.005–0.1 Hz), censoring data points with excessive motion (root mean square of three-dimensional [3D] displacement > 0.3 mm), and smoothing using a Gaussian kernel (full width at half maximum = 6 mm). Additionally, six affine motion parameters, signal from a ventricular region of interest, and signal from an area centered in the white matter were regressed out of the dataset. The whole-brain global signal was not removed, as doing so can result in spurious anti-correlation (Fox et al., 2009; Murphy et al., 2009). Individuals’ fMRI images were spatially co-registered to their high-resolution anatomical images in a linear transform using AFNI’s “align_epi_anat.py” command, and then further transformed to the Talairach and Tournoux template brain (Talairach and Tournoux, 1988) to ensure spatial normalization in the group-level analysis.

### 2.5 Functional connectivity mapping and analysis

For each subject, a mask covering the tumoral resection area was identified on structural MRI in AFNI. To generate the mask, the processed structural MRI in T1 contrast was copied into 3D Slicer^1^ (Kikinis et al., 2013). In 3D Slicer, the draw tool was used to outline a two-dimensional (2D) mask for each slice manually. Once the mask for all slices had been outlined and filled, the area between slices was filled to generate a 3D mask volume. The authors HY and DM produced the mask in 3D Slicer, which was reviewed and verified by the author and neurosurgeon IFD. The mask was copied into AFNI by saving the 3D Slicer mask as a NIFTI volume, which was then resampled using the processed fMRI volume as a template. To generate a mask encompassing only the perilesional tissue, the resection mask was dilated by three neighboring voxels. Considering that 1 cm is the margin typically preserved in surgical resection to protect eloquent functions (Hervey-Jumper and Berger, 2016), 3 voxels at an in-plane resolution of 3.75 × 3.75 mm will approximate the margin. Then the inner masked area was subtracted from the dilated mask, resulting in the perilesional mask. In addition, to compare with the contralateral tissue, a contralateral mask was generated by mirroring the perilesional mask across the midline of the brain in conjunction with the whole-brain mask to exclude non-brain tissue. All subjects’ contralaterally masked regions were visually inspected on the structural images and were verified to be free of tumor infiltration, edema, or significant mass effect. A mask of the whole brain, subtracting the resected area, was also generated, so that the following analysis steps excluded the blank voxels of the surgical cavity and any non-brain voxels.

**Figure 1:**
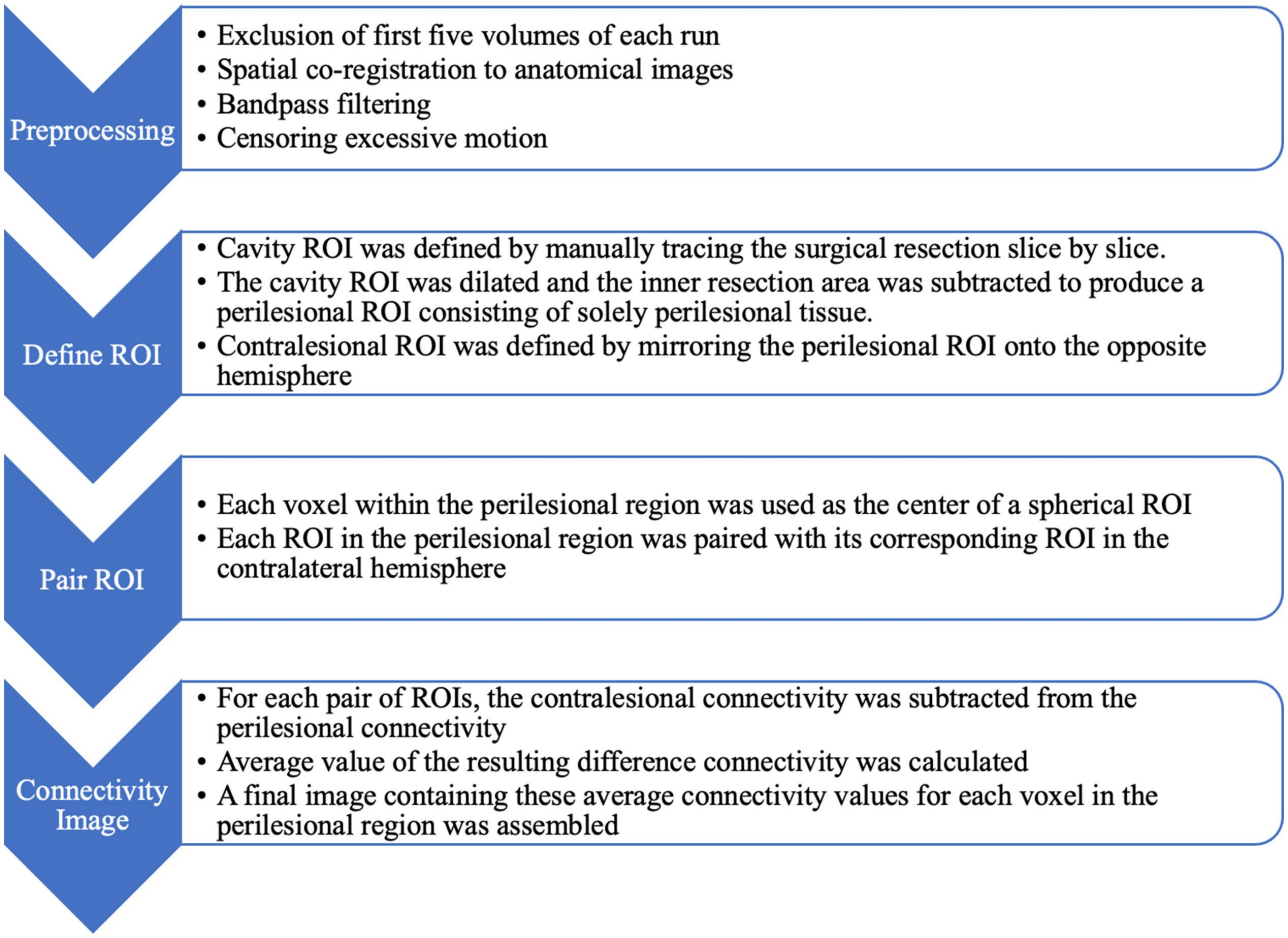
A schematic diagram of data processing steps.

To quantify the functional connectivity, a seed-based ROI approach was used. For each mask, the coordinates of the masks were used to select each voxel within the perilesional region as the seed. An ROI was created as a sphere centered at the seed with a 5-mm radius and in conjunction with the whole-brain mask. Considering that 5 mm is the radius typically selected in resting-state functional connectivity studies (Power et al., 2011) and also considering that 1 cm is a margin typically preserved in surgical resection to protect eloquent functions (Hervey-Jumper and Berger, 2016), the 5-mm radius was chosen at a resolution that matches the margin considered in surgical procedures. For each seed within the perilesional region, the average Blood Oxygen Level Dependent (BOLD) time series within the seeded ROI was extracted and correlated with every voxel in the cavity-excluded brain mask to determine the overall connectivity value for that seed. The resulting Pearson’s correlation coefficients were then converted into *z*-scores using Fisher’s transform. The same process was repeated for the seeds within the contralateral mask that were paired with their counterparts in the perilesional region. For each voxel, contralateral connectivity was subtracted from perilesional connectivity, and the resulting connectivity difference map was averaged. The average value was paired with the coordinates of the perilesional voxel with which it was associated. A final heatmap was created using the AFNI *undump* function. This map, namely the connectivity difference map, was displayed over the perilesional region, with each voxel’s value set to the connectivity difference between that voxel’s seed and its corresponding seed in the contralateral region. Figure 1 shows a schematic of the data analysis process described.

### 2.6 Statistical analysis

We evaluated whether fMRI connectivity acquired immediately after surgery could predict patients’ survival time. We divided the patients into two subgroups based on their median survival time: those with survival greater than the median survival time, and those with survival less than the median survival time. Then we tested whether the connectivity values differed between these two groups. A Kolmogorov–Smirnov test was used to study the distribution of each variable. When the data did not follow a normal distribution, a Mann–Whitney U-test was used for testing the difference between two variables.

Furthermore, we assessed the association between KPS changes (i.e., the scores at 6-month follow-up subtracting pre-surgical scores). First, considering that our study was primarily aimed at determining whether the connectivity metrics contain any predictive information, we used a univariate regression model on the connectivity (predictor variable) and KPS (outcome variable), and we calculated Spearman’s correlation coefficient *ρ* between the connectivity and KPS. In addition, we considered a multivariate generalized linear regression model with the KPS as outcome variable, then connectivity, age, sex, tumor grade, and tumor volume as predictor variables. Due to the small sample size available in our study, no other predictor variables were included. ANOVA evaluated whether any of the predictors were significant. A significance level of 5% (*p* < 0.05) was accepted in all cases.

### 2.7 Machine learning prediction

In addition to the above regression analysis, which produced interpretable results regarding the connectivity metrics, we also used a machine learning model to predict survival time and KPS outcomes. Specifically, a support vector machine (SVM) model with a Gaussian kernel was chosen, since SVM has been shown to have reliable performance in a small sample of a dataset. The connectivity difference values from the perilesional and contralesional regions were used as features. The prediction was set as binary: greater than the median survival time (15 months) was “1” and less than the median survival time (15 months) was “0.” The leave-one-out cross-validation (LOOCV) was performed to evaluate prediction performance. Accuracy was measured based on the classification results of the validation. True positive, true negative, false positive and false negative were calculated as well.

Additionally, we used the SVM regression model to predict the KPS scores. Again, the connectivity difference values between the perilesional and the contralesional regions were used as features. The leave-one-out cross-validation was performed to evaluate prediction performance. The Spearman’s rank correlation coefficient between the true KPS and the predicted KPS was calculated. Since the SVM with a Gaussian kernel is a non-linear model, we included a linear regression with leave-one-out cross-validation to compare its predictive performance and its robustness to sample outliers.

## 3 Results

### 3.1 Clinical characteristics

Thirteen patients participated in the study protocol. One subject was excluded from analysis due to incomplete brain coverage of fMRI data. The remaining 12 participants included eight women and four men, with an age at the time of surgery of 46.83 ± 12.17 years (range: 28–66 years). Nine subjects had Grade IV gliomas, two had Grade II gliomas, and one had a Grade I glioma. Since the purpose of the study is to evaluate the prognostic capacity of fMRI acquired within 3 days postsurgical, our analysis included all subjects. Then we repeated the analysis in the subsets of Grade IV subjects and malignant tumor subjects only. Across all subjects, the survival time was 39.55 ± 34.67 months from diagnosis (range: 0–71.88 months). The median survival time was 14.4 months, but only 6 of 12 survived longer than 1 year after resection. Six subjects underwent awake resection, only three of whom had a Grade IV glioma. Subject characteristics are displayed in Table 1.

**Table 1:** Patient demographics and clinical characteristics.

Characteristic	Number of subjects (Total: *N* = 12)
Age at time of surgery (year)	46.83 ± 12.17
Sex	
Male	4
Female	8
Tumor location	
Frontal	2
Parietal	1
Temporal	7
Basal ganglia	1
Frontotemporal	1
WHO classification	
Grade I	1
Grade II	2
Grade III	0
Grade IV	9

### 3.2 Brain connectivity and survival time

**Figure 2:**
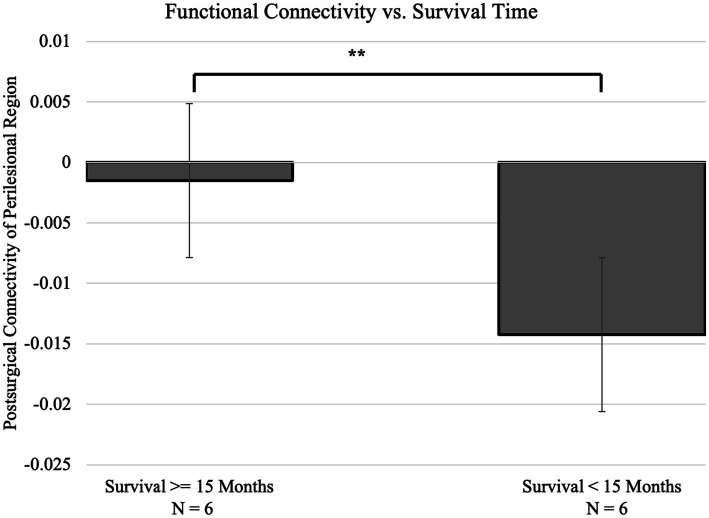
Comparison of perilesional connectivity between two groups of glioma patients who survived either more than 15 months (*N* = 6) or fewer than 15 months from diagnosis (*N* = 6). The two groups differed significantly in connectivity, with the group with shorter survival time showing a smaller difference in perilesional connectivity (*p* = 0.0016 and Cohen’s *d* = 2.74). **p < 0.01.

**Figure 3:**
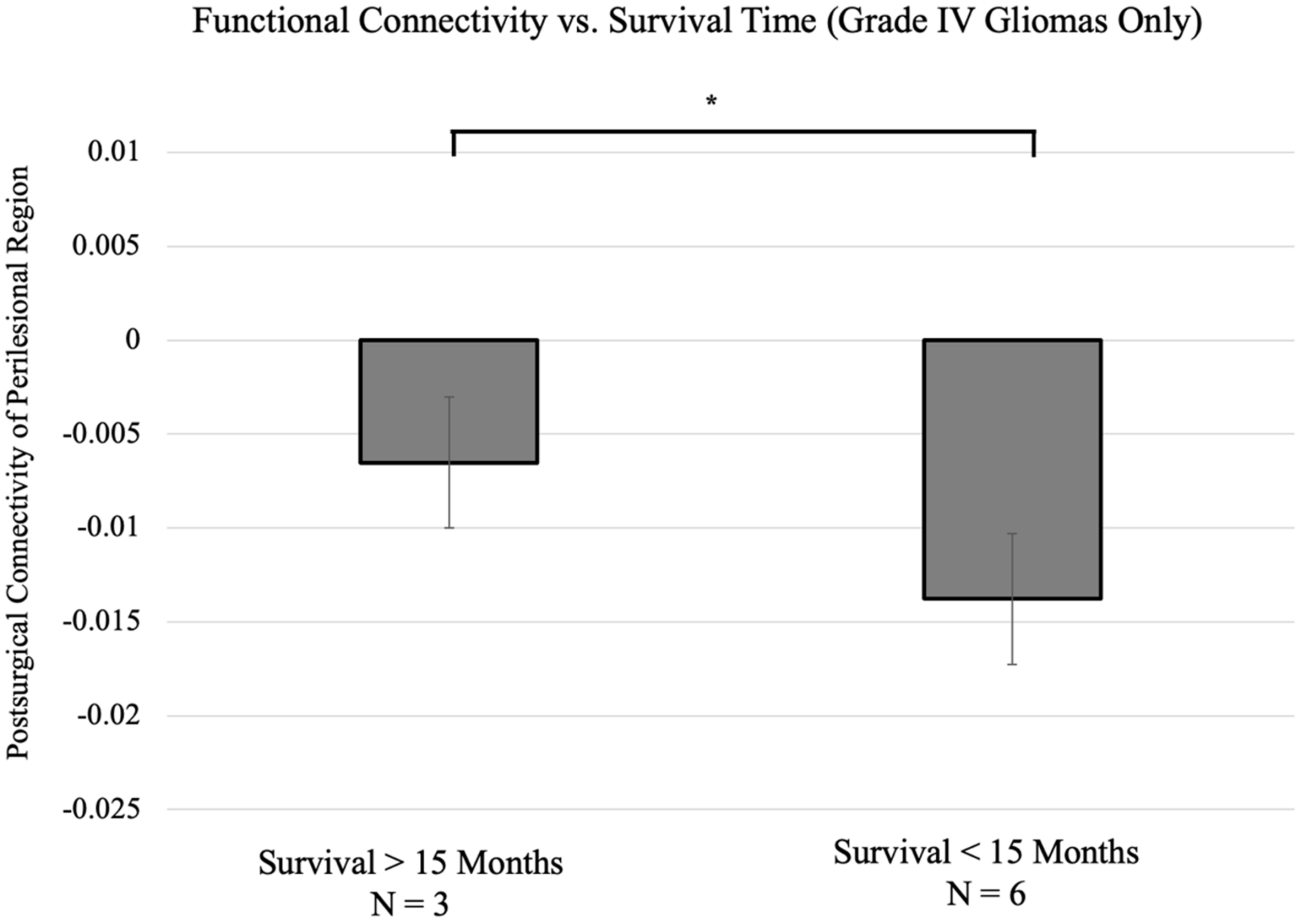
Comparison of perilesional connectivity between two groups of grade IV only glioma patients surviving either greater than 15 months (*N* = 3) or fewer than 15 months from diagnosis (*N* = 6). The two groups differed significantly in connectivity, with the group with the group with shorter survival time showing a smaller difference in perilesional connectivity (*p* = 0.02 and Cohen’s *d* = 1.90). *p < 0.05.

We divide the patients into two subgroups: six of the patients survived less than the median time (14.4 months), and the remaining six patients survived more than the median time (this subgroup includes four subjects who were still living at the time of data collection). The division is the same as dividing the patients by whether they survived 15 months. With this division, a significant difference in connectivity of the perilesional region compared to the corresponding contralateral region was presented in subjects who survived fewer than 15 months from diagnosis compared to those surviving 15 months or longer (Figure 2). Among the 6 subjects who survived fewer than 15 months, the survival time was 4.80 ± 4.48 months, and the perilesional connectivity difference was −0.0138 ± 0.0047. Among the six subjects who survived for 15 months or longer, the survival time was 53.53 ± 34.64 months, and the perilesional connectivity difference was −0.0042 ± 0.0038. With a positive result for the Kolmogorov–Smirnov test on the variables, a two-tailed Mann–Whitney U-test showed a significant difference in the connectivity (*p* = 0.0016 and Cohen’s *d* = 2.74). Since the tumor grade has been shown to predict survival time and the majority of patients were Grade IV, we repeated the analysis in patients with Grade IV and compared the two subgroups with longer and shorter survival times. Importantly, this difference remained significant in the subset of subjects with only Grade IV gliomas (*p* = 0.02 and Cohen’s *d* = 1.90) (Figure 3). We also repeated the analysis in patients with malignant glioma only (excluding one subject with Grade I tumor resection), and the difference was still significant (*p* = 0.0043 and Cohen’s *d* = 2.32).

### 3.3 Brain connectivity and functional outcome

**Figure 4:**
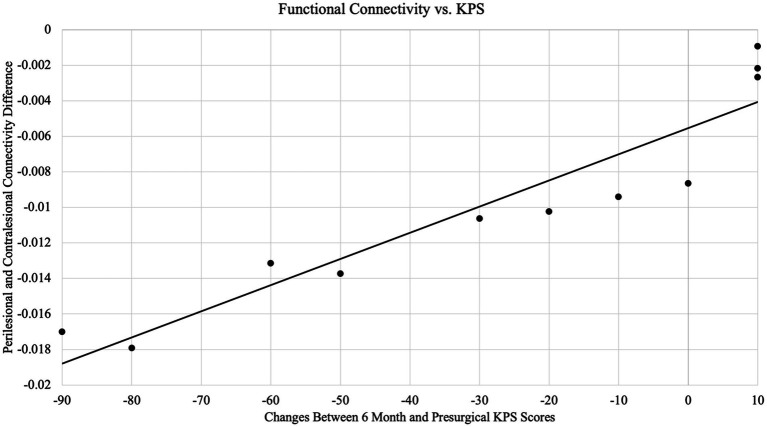
Association between functional connectivity and Karnofsky performance scale (KPS) scores. There is a significant positive correlation between neurological outcome changes in 6 months postoperative and functional connectivity differences between perilesional and contralesional regions (*ρ* = 0.97, *p* < 0.001).

To examine whether immediate postsurgical scans can predict the extent of functional recovery following surgery, we evaluated the association between connectivity and KPS score differences between the preoperative assessment and 6-month follow-up visit (Figure 4). One subject did not attend the 6-month follow-up, so only 11 subjects were included in this analysis. Four subjects had an improved or unchanged KPS score at the 6-month follow-up as compared to their preoperative evaluation. The remaining seven subjects had a decline in KPS over the same period. The Spearman’s correlation coefficient between the difference in KPS at 6 months compared to the preoperative assessment and the connectivity difference between the perilesional and contralateral regions was 0.97 (*p* < 0.001). Subjects exhibiting largely decreased connectivity in the perilesional region had more substantial declines in functional status than those exhibiting more neutral changes in perilesional connectivity. In a multivariate generalized linear regression model “KPS ~ 1 + Connectivity + Age + Sex + WHO Grade + Tumor Volume,” our analysis found that the model significantly predicted KPS (*p* = 0.0056) with *R*-squared: 0.97 and Adjusted *R*-Squared: 0.92. Among all variables, only the connectivity was a significant predictor (*p* = 0.0017) with CI = [5.6 × 10^3^, 1.2 × 10^4^]. None of the other predictors were significant (WHO grade approached significance with *p* = 0.072; age, sex, and tumor volume have *p* > 0.1). Additionally, in the subset of Grade IV patients only, the Spearman’s correlation coefficient between connectivity values and KPS changes was 0.95 (*p* = 0.001). A multivariate model in Grade IV patients only significantly predicted KPS (*p* = 0.01), with *R*-squared: 0.97 and Adjusted *R*-Squared: 0.93. The connectivity value among Grade IV patients alone remained the only significant predictor of KPS changes (*p* = 0.003), whereas the other variables (age, sex, and tumor volume) were not significant (all *p* > 0.1). Furthermore, in the subset of malignant subjects (excluding the subject with Grade I resection), the connectivity value remained significantly associated with KPS changes (*ρ* = 0.97, *p* < 0.001).

### 3.4 Representative case of sub-median survival

**Figure 5:**
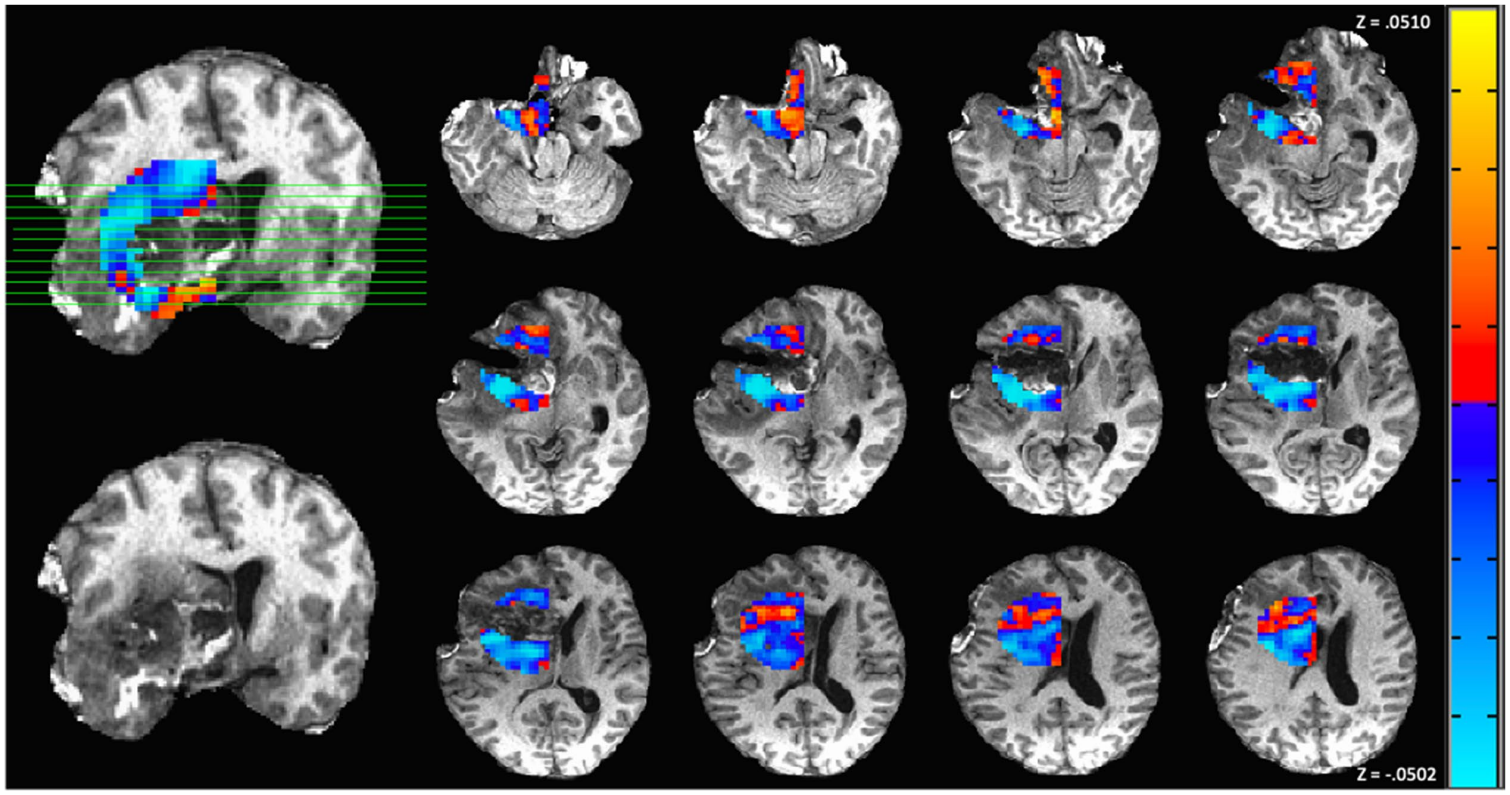
Functional connectivity map from one representative subject with left frontotemporal World Health Organization (WHO) grade IV glioblastoma. In these images, red represents an increase in functional connectivity of the perilesional region compared to the corresponding contralateral region, and blue represents a relative decrease. This patient is a representative example of primarily decreased connectivity. Before surgery, the patient presented a Karnofsky Performance Status (KPS) score of 60. However, the patient did not survive to the 6-month postsurgery benchmark, indicating a KPS change of −60.

The above group results indicated that patients exhibiting largely decreased connectivity in the perilesional region had shorter survival time and more substantial declines in functional status than those exhibiting more neutral changes in perilesional connectivity. A representative subject with sub-medial survival is shown in Figure 5. The subject was a 60-year-old woman who underwent surgical resection of a Grade IV glioblastoma in the frontotemporal lobe. The subject’s preoperative KPS score was 60, and the immediate postoperative KPS score was 60. The connectivity map of postsurgical scans shows an overall decrease in connectivity in the perilesional area relative to the contralesional regions, suggesting a status of weakened connections at the postsurgical time point. This subject survived for under 3 months from diagnosis. At 6 months postoperative, however, the subject was deceased, leading to a KPS score of zero. In this case, the perilesional connectivity of below-zero values suggested a prediction of sub-median survival.

### 3.5 Representative case of above-median survival

**Figure 6:**
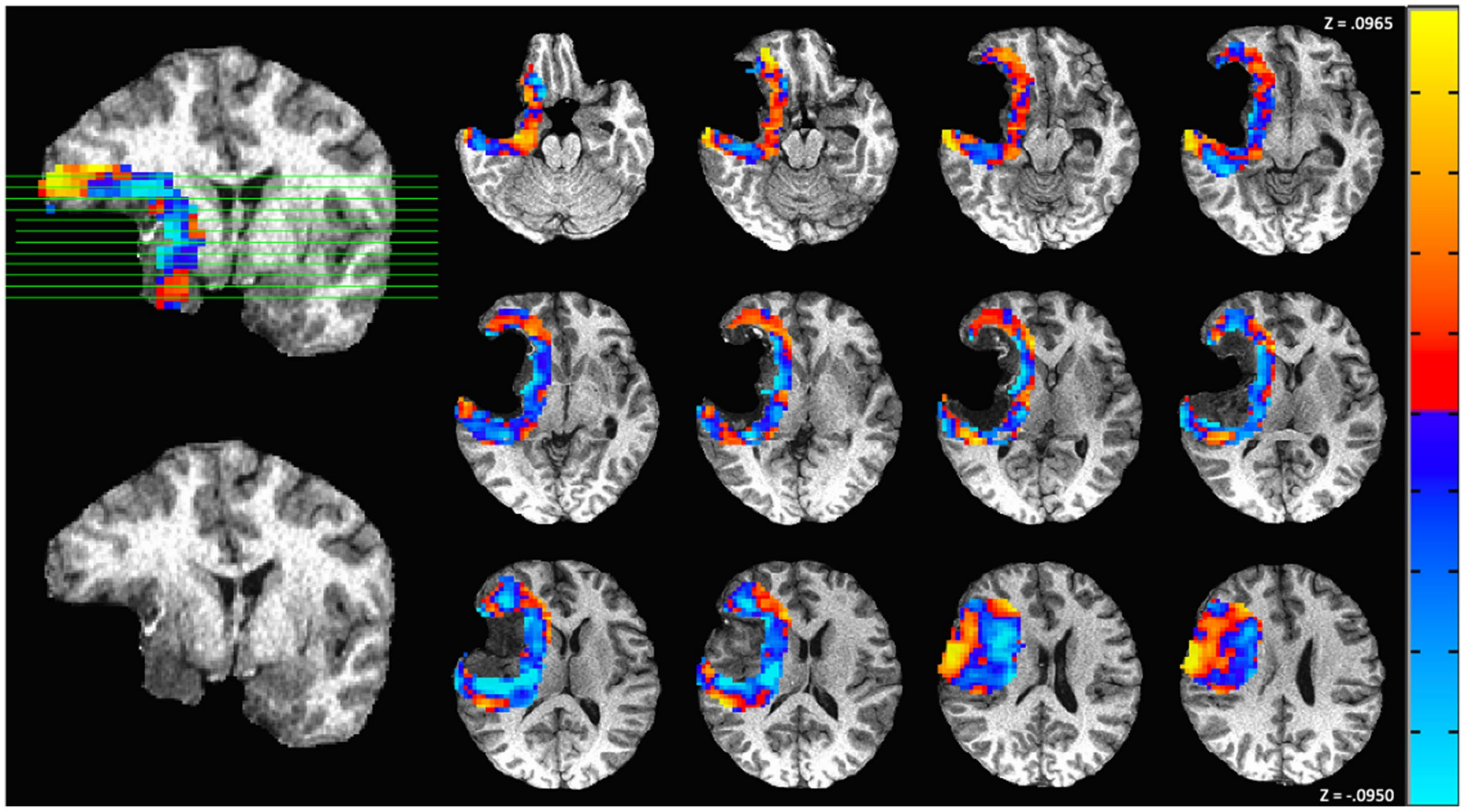
Functional connectivity map from another representative subject with left temporal World Health Organization (WHO) Grade IV glioblastoma. Red represents an increase in functional connectivity of the perilesional region compared to the corresponding contralateral region, and blue represents a relative decrease. This patient had a more equal distribution of increases and decreases in connectivity. Prior to surgery, the patient presented a Karnofsky Performance Status (KPS) score of 90. At the 6-month postsurgery benchmark, the patient achieved a KPS score of 90, indicating a 0-point difference. The patient survived 4 years after diagnosis.

The results of another representative subject with above-median survival are shown in Figure 6. This subject was a 32-year-old woman who underwent surgical resection of a Grade IV glioblastoma in the temporal lobe. The subject’s preoperative KPS score was 90, but it decreased to 80 at the immediate postoperative assessment. The connectivity map of postsurgical scans presents a mixture of decreases and increases of connectivity in the perilesional area relative to the contralesional regions. While the perilesional decreases indicate a status of weakened connections at the postsurgical time point, the relative increases suggest enhanced connections, possibly due to the recruitment of perilesional tissues during the recovery process. At the 6-month follow-up, the subject’s KPS score returned to 90, indicating recovery of functions lost from resection. This subject survived 4 years from diagnosis.

### 3.6 Machine learning prediction

**Figure 7:**
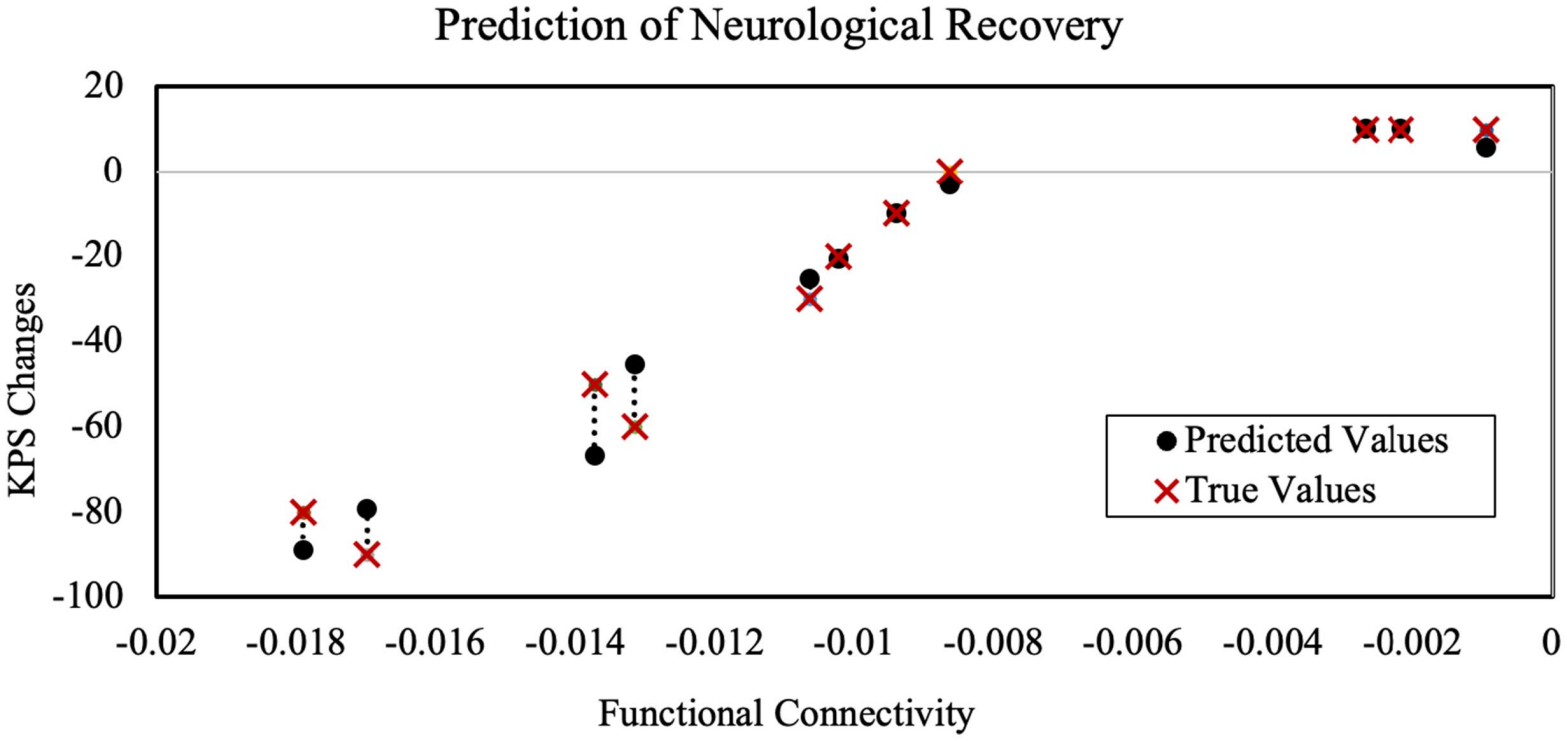
Comparison between true and predicted Karnofsky performance scale (KPS) changes based on functional connectivity using a support vector machine based on leave-one-out cross-validation. The Spearman’s correlation coefficient between true values and predicted values is 0.97 (*p* < 0.001).

Based on the functional connectivity values, the SVM was able to predict the survival time with an LOOCV accuracy of 92%. Specifically, for predicting a greater than 15-month survival time, the true positive rate was 83%, the false negative rate was 17%, the true negative rate was 100%, and the false positive 0%. Furthermore, the SVM regression model was able to predict the KPS score changes with an absolute error of 5.84 ± 6.08. As shown in Figure 7, the resulting Spearman’s correlation coefficient between the true and predicted KPS was 0.97 (*p* < 0.001). In comparison, the linear regression model with leave-one-out cross-validation yielded larger absolute error (12.48 ± 8.26), and the Spearman’s correlation coefficient was the same (*ρ* = 0.97, *p* < 0.001).

## 4 Discussion

In the current proof-of-concept study, we investigated the prognostic value of postsurgical fMRI data in glioma patients obtained within an acute time window of 3 days after surgery and before discharge. We developed an analytic method to quantify the postoperative whole-brain functional connectivity from the postsurgical scans. Especially, the functional connectivity pattern of perilesional regions with regard to their corresponding contralateral regions was found to be related to the patients’ survival time and their neurological recovery at 6-month follow-up. Our results revealed that the functional connectivity significantly differed between the subgroup of patients with survival time greater than 15 months and those who did not. Moreover, the values of functional connectivity were significantly correlated with changes in KPS at 6 months after surgery. Using a machine learning model, functional connectivity predicted the neurological outcomes with high accuracy. Our findings strongly suggest that fMRI acquired acutely after surgery contains long-term prognostic information.

Structural and functional imaging has been used to understand the altered organizations in patients with gliomas (Lamichhane et al., 2023; Patronas et al., 1985; Rau et al., 2014; Yan et al., 2017). More recently, resting-state fMRI is increasingly used for its functional relevance as well as wide access. Most prior work reported presurgical findings (Stoecklein et al., 2020; Tarapore et al., 2012; Zimmermann et al., 2024), a few studies compared the presurgical and postsurgical data (Daniel et al., 2021), including one of our own works (Huang et al., 2025). Our study has extended the prior findings and added new knowledge regarding the acute postsurgical stage. Studies of presurgical scans revealed that tumoral and peritumoral regions present abnormal connectivity compared to the contralateral regions, and this phenomenon has been observed using magnetoencephalography (Tarapore et al., 2012; Zimmermann et al., 2024) or fMRI (Daniel et al., 2021; Noll et al., 2021; Stoecklein et al., 2020). When compared with the healthy control data, the tumoral/peritumoral connectivity values spread out a wide range from being normal to abnormal, but the contralateral connectivity mimics a healthy distribution (Daniel et al., 2021; Stoecklein et al., 2020; Zimmermann et al., 2024), which indicates that the difference between tumoral and contralateral site could be an index of abnormality. In line with presurgical findings, the results of the current study showed that functional connectivity in postsurgical fMRI scans of all 12 subjects exhibited decreased connectivity in the perilesional region as compared to the mirrored contralateral region. Such decreases of connectivity in areas surrounding tumor resection after surgery are similar to those decreases seen in areas at the tumor boundary before surgery (Tarapore et al., 2012; Zimmermann et al., 2024). Alterations in networks have been reported when comparing presurgical images with postsurgical images (Huang et al., 2025; Noll et al., 2021), but our findings have filled the gap regarding the acute postsurgical stage.

Our analytic approach differs from many previous studies, and our motivation is driven by clinical relevance and the fact that the perilesional tissue is a critical area for predicting postoperative survival time and functional outcome recovery. While often the whole-brain functional connectivity was examined (Stoecklein et al., 2020; Tarapore et al., 2012; Zimmermann et al., 2024) and certain studies focused on connectivity between mirroring/homotopic brain regions (Daniel et al., 2021), presurgical images commonly present the tumoral and peritumoral areas with a mixture of increased and decreased connectivity than the contralateral homologue area. In contrast, our analysis of postsurgical images considered connectivity between the perilesional area and the whole brain (rather than homotopic connectivity alone). The perilesional tissue in postsurgical scans approximates the location of peritumoral tissue in the presurgical scans, but their activities still differ. Our findings suggest that the weakened connectivity surrounding the tumor is pathological and is preserved from the presurgical to the postsurgical stages. Both surgical-induced inflammation and plasticity of the brain in response to tumors can alter the connectivity of perilesional tissue (Carpentier et al., 2001; Nieberlein et al., 2023; Tarapore et al., 2012; Vlieger et al., 2004). In gliomas, the decline in cognitive function would be reflected in in altered functional connectivity. Meanwhile, the plasticity of the brain allows for the restructuring or reorganization of the neural networks governing functions (Burke and Barnes, 2006; Cargnelutti et al., 2024; Kourosh-Arami et al., 2021). This phenomenon allows for the brain to restructure in response to glioblastoma, so some functions of the glioma area may be redistributed to other regions of the brain and not be lost through resection (Tarapore et al., 2012). Importantly, our findings have extended the discovery of altered connectivity to the postsurgical stage and demonstrated its predictive value in terms of prognostic information.

Despite the overall abnormality in the perilesional tissue, the functional connectivity presents individual patterns across the patients (representatives shown in Figures 5 and 6). Our analysis explored these connectivity patterns and found that they are indicative of glioma patients’ functional recovery, as indexed by changes in KPS. A greater decrease in the perilesional region is associated with worsened neurological outcome—less survival time and more deficits. Our finding is consistent with the trends reported by presurgical studies, which have demonstrated that the tumoral/peritumoral connectivity is predictive of the neurological outcome, in terms of both the survival time (Daniel et al., 2021; Stoecklein et al., 2020; Zimmermann et al., 2024) and the long-term neurological deficits (Tarapore et al., 2012). The relative increases and decreases of connectivity in the perilesional region than the contralateral site could be attributed to the inflammatory responses to surgery, as well as the retention of perilesional function (Tarapore et al., 2012). While our method used the contralateral area as a reference point to calibrate the connectivity within the peri-lesional region, the symmetry between homologous regions in brain tumor patients is subject to subtle changes after surgical resection, especially in patients with large tumor volumes. Nonetheless, the connectivity has shown strong predictive capacity despite subtle changes in homologous contralateral regions. Despite a small sample size, our analysis has found a very strong correlation (*ρ* = 0.97) between the KPS and functional connectivity, and the correlation in the subset of Grade IV-only subjects remains strong (*ρ* = 0.95). The high correlation could be attributed to the fine delineation of perilesional areas in our method as well as our analysis has calculated the whole-brain connectivity regarding the perilesional tissue, whereas prior studies considered the whole-brain connectivity or somatotonic connectivity without refining the connectivity to the tumoral boundaries (Stoecklein et al., 2020; Daniel et al., 2021).

Furthermore, we hypothesized that further decreased connectivity within the perilesional region would be indicative of shortened survival time postsurgery. To test this hypothesis, we divided the patients into two subgroups according to the median survival time, that is, 14.4 months, which was very close to the reported median survival time for glioma of all grades—15 months. Dividing our sample by 15 months or 14.4 months yielded the same subgroups, so we chose 15 months, consistent with other benchmark studies. Indeed, our analysis found that patients who survived less than 15 months beyond surgery exhibited more severe decreases in perilesional connectivity than patients who survived longer after surgery (*p* = 0.0016 and Cohen’s *d* = 2.74). Notably, this difference was still significant when considering only grade IV glioma (*p* = 0.02 and Cohen’s *d* = 1.90) or considering malignant gliomas only and excluding the Grade I (*p* = 0.0043 and Cohen’s *d* = 2.32). In predicting survival time, our analysis achieved an accuracy of 92% in classifying whether patients had surpassed a survival time of 15 months. Despite a small sample, our accuracy is at par with other reports in larger samples based on a combined structural and functional scans CT + fMRI: 90.6% by (Luckett et al., 2023), DTI + fMRI: 89.9% (Nie et al., 2016), while higher than using CT only (83% (Lamichhane et al., 2023) or fMRI alone (71.9% by (Lamichhane et al., 2021). Survival of glioma can be reliant on retention of cognitive function, which is both reflective of the severity. Given a controlled EOR, increased neurological morbidity coincides with decreased function and shortened survival (Sanai and Berger, 2018). Thus, excessive decreases in perilesional connectivity suggest a connection to shortened survival time.

These findings, which promote retention of expected connectivity values in perilesional tissue for retention of overall function, are vital to a greater understanding of surgical planning to limit neurological morbidity and extend survival. In combination with previous studies examining the relationship between long-term functional outcome, survival, and perilesional/lesional connectivity, understanding of functional connectivity in glioblastoma patients may be implemented to increase preoperative planning and boundary determination. In place of intraoperative mapping, which can be limited by health risks and increased craniotomy (Hervey-Jumper and Berger, 2016; Vlieger et al., 2004), preoperative mapping to define boundaries based on functional imaging could prove to be safer and more effective. Rather than stimulus-based functional mapping that focuses on sensorimotor, language, and vision function (Vlieger et al., 2004), a lesional/perilesional to contralateral comparison of overall functional connectivity could be beneficial for overall retention of function and possibly extended survival. Tarapore et al. showed that resecting tumoral tissues with increased connectivity than the contralateral area is associated with long-term neurological deficits, while the resection of decreased connectivity with no deficit; their analysis has suggested a resection strategy based on the individual patient’s connectivity map that was produced, resecting the area with decreased connectivity while avoiding the area with increased connectivity. Importantly, our study further suggests that maximizing the resection area with decreased connectivity might lead to an optimal outcome.

Though this study presents novel functional imaging in a postsurgical cohort of glioma patients, it is limited in several ways. Primarily, its small sample size limits the extent of variation that could be examined. Brain gliomas exhibit significant heterogeneity (Yabo et al., 2022), so a sample of only 12 subjects does not capture the variability in size, location, severity, and molecular markers, such as isocitrate dehydrogenase (IDH) mutation status, that can occur. Although the sample size was small, our results remained significant across the whole group of glioma subjects, the subgroup of Grade IV only subjects, and the subgroup of malignant-only subjects. While the preliminary Grade of the tumor can be classified intraoperatively, the full report typically takes up to a few weeks. The prognostic information regarding the long-term neurological outcomes that are obtainable acutely after surgery and before patient discharge can still be very valuable in facilitating the physicians, patients, and care providers in the postsurgery management. In particular, resting-state fMRI can be added to the already scheduled MRI scans and efficiently acquired within the clinical workflow. Nonetheless, while the current study suggests the merit of fMRI in providing prognostic information, it remains unclear to what extent it contributes additionally to the existing knowledge on prognostic predictors based on surgery and clinical characteristics. Going forward, similar investigations would benefit from increased recruitment, longitudinal imaging, and comprehensive data that incorporate multimodal imaging and molecular markers to further evaluate the predictive value of the functional connectivity. Another technical limitation of the current study is the use of a 1.5 T MRI scanner, which has a lower signal-to-noise ratio (SNR) than the modern standard of a 3.0 T scanner. While the results achieved are robust, subtle or smaller-scale functional changes may have been underestimated or missed due to the lower SNR, and it remains to be seen whether similar discoveries on a 3.0 T MRI can be replicated. Furthermore, in addition to the strength of functional connectivity being a predictive biomarker, other metrics, such as graph theory metrics that quantify the integration of the perilesional region with large-scale networks, especially at the whole-brain level, can provide a more holistic understanding of postoperative brain reorganization and warrant future investigations.

## 5 Conclusion

In the current proof-of-concept study, we investigated the prognostic value of postsurgical fMRI data in glioma patients obtained within an acute time window of 3 days after surgery and before discharge. Our analysis has reported an analytical method for quantifying the postoperative whole-brain functional connectivity. In all patients we examined, the perilesional tissue surrounding the tumor resection exhibited overall decreased functional connectivity compared with the contralateral homologue area. Importantly, the functional connectivity value was a strong indicator of the extent of neurological recovery (*ρ* = 0.97, *p* < 0.001) and overall survival time (Cohen’s *d* = 2.74 and *p* < 0.001). Our findings are in concurrence with previous studies that examined functional connectivity in patients with glioma, however, strictly before surgery. Furthermore, based on machine learning models, connectivity as features can predict survival time with an accuracy of 92% and also predict KPS changes at 6-month follow-up with a Spearman’s correlation coefficient of 0.97 and an absolute error of 5.84 ± 6.08. These neuroimaging findings suggest that the functional organization of brain networks plays an important role in recovery after surgical resection of gliomas. Our study suggests that fMRI acquired acutely after surgery may contain long-term prognostic information.

## Data Availability

Data used in this study are not publicly available due to data sharing restrictions from the IRB but are available from the corresponding author through a data use agreement upon reasonable request. Software source code supporting the conclusions of this article is available from the corresponding author upon reasonable request.

## Footnotes

1
http://www.slicer.org/
